# Diabetes Distress and Self-Reported Health in a Sample of Alabama Medicaid-Covered Adults Before and During the COVID-19 Pandemic

**DOI:** 10.3389/fcdhc.2022.835706

**Published:** 2022-05-10

**Authors:** Alesha C. Amerson, Lucia D. Juarez, Carrie R. Howell, Emily B. Levitan, April A. Agne, Caroline A. Presley, Andrea L. Cherrington

**Affiliations:** ^1^ School of Medicine, University of Alabama at Birmingham (UAB), Birmingham, AL, United States; ^2^ Division of Preventive Medicine, University of Alabama at Birmingham (UAB), Birmingham, AL, United States; ^3^ Department of Epidemiology, School of Public Health, University of Alabama at Birmingham (UAB), Birmingham, AL, United States; ^4^ Department of Nutrition Sciences, University of Alabama at Birmingham (UAB) Diabetes Research Center, Birmingham, AL, United States

**Keywords:** diabetes, self-reported health, medicaid, COVID, survey

## Abstract

Temporary closures of outpatient health facilities and transitions to virtual care during the COVID-19 pandemic interrupted the care of millions of patients with diabetes contributing to worsening psychosocial factors and enhanced difficulty in managing type 2 diabetes mellitus. We explored associations between COVID time period and self-reported diabetes distress on self-reported health among a sample of Alabama Medicaid-covered adults with diabetes pre-COVID (2017-2019) and during-COVID (2020-2021).

**Method:**

In this cross-sectional study, we surveyed a population-based sample of adults with type 2 diabetes covered by the Alabama Medicaid Agency. Participants were dichotomized into pre-COVID (March 2017 to October 2019) vs during-COVID (October 2020 to May 2021) groups. Participants with missing data were removed from analyses. We assessed diabetes related stress by the Diabetes Distress Scale. We measured self-reported health using a single item with a 5-point Likert scale. We ran logistic regressions modeling COVID time period on self-reported poor health controlling for demographics, severity of diabetes, and diabetes distress.

**Results:**

In this sample of 1822 individuals, median age was 54, 74.5% were female and 59.4% were Black. Compared to pre-COVID participants, participants surveyed during COVID were younger, more likely to be Black (64.1% VS 58.2%, p=0.01) and female (81.8% VS 72.5%, p<0.001). This group also had fewer individuals from rural areas (29.2% VS 38.4%, p<0.001), and shorter diabetes duration (7 years VS 9 years, p<0.001). During COVID individuals reported modestly lower levels of diabetes distress (1.2 VS 1.4, p<0.001) when compared to the pre-COVID group. After adjusting for demographic differences, diabetes severity, and diabetes distress, participants responding during COVID had increased odds of reporting poor health (Odds ratio [OR] 1.41, 95% Confidence Interval [CI] 1.11-1.80).

**Discussion:**

We found respondents were more likely to report poorer health during COVID compared to pre-COVID. These results suggest that increased outreach may be needed to address diabetes management for vulnerable groups, many of whom were already at high risk for poor outcomes prior to the pandemic.

## Introduction

The coronavirus disease of 2019 (COVID-19) pandemic had extensive impact in the United States causing over 700,000 deaths and sparked new fears of economic turmoil and social isolation (1). The US underwent several protective measures including temporary closure of public facilities, suspending external travel, and nightly curfews to prevent the spread of coronavirus (2).

These changes posed new barriers to care for patients with chronic disease including closing of outpatient clinics, decreased inpatient capacity, staff shortage, and medicine shortage. During COVID, many patients were unable to afford medicines or access transportation resulting in delay in seeking care (3, 4), For patients with Diabetes Mellitus (DM), these experiences could contribute to worse disease management and elevated diabetes distress, defined as an emotional state where people experience feelings such as stress, guilt, or denial that arise from living with diabetes and the burden of self-management (5, 6). Increased diabetes distress is closely linked to poor glycemic control in adults with DM (7).

The “Diabetes Belt,” a term coined by the Centers for Disease Control (CDC), refers to the southeastern region of the United States where prevalence of DM is disproportionately high (> 11%) as are rates of diabetes-related complications and mortality (8). Notably, this region has a greater percentage of African Americans (23.8% compared to 8.6%) and higher rates of poverty compared to the rest of the country (9). Centrally located within the Diabetes Belt, Alabama is the 6th poorest state in the US with over 37% of the population living at or below 200% federal poverty level (7). Limited studies have reviewed the effects of the COVID pandemic on this population. The compounding high burden of disease with limited financial resources may further complicate diabetes management and may worsen diabetes distress.

Evidence also suggests that COVID protective measures may have had significant impact on individuals physical and mental health, with some populations experiencing higher rates of anxiety, depression, and perceived stress during the pandemic (10). Moreover, individuals who were directly impacted by COVID reported declines in self-rated health (11). Lower self-rated health and higher levels of mental distress are associated with poor health management, increased hospitalizations, and increased mortality in patients managing chronic disease (12).

In this study, we examined the relationship between diabetes distress, depressive symptoms, diabetes management self-efficacy, and perceived stress with self-reported health in Alabama Medicaid-covered adults with diabetes between COVID time periods (prior to vs during).

## Methods

### Study Design and Population

We conducted a cross-sectional survey within the Alabama Care Plan study. Briefly, the Alabama Care Plan (ACP) was an observational study of the quality of care of adults with diabetes covered by Alabama Medicaid. The Alabama Care Plan study enrolled a population-based sample of adults with type 1 or 2 diabetes who were covered by Alabama Medicaid between March 2017 and May 2021. Medicaid eligibility for adults in Alabama includes parents of minor children with incomes at or below 18% of Federal Poverty Level (FPL) and adults with disability eligible for the Supplemental Security Income (SSI) program (13). As part of the ACP study, a survey was conducted among a sample of Medicaid-covered adults with diabetes to assess patient-reported outcomes and satisfaction with care.

The current study examines survey responses before and during the COVID pandemic. Adults were eligible if they met the following criteria: age 19 to 64 years old, covered by Medicaid for the prior 12 months, and were diagnosed with diabetes, defined by the presence of at least one inpatient or two outpatient International Classification of Diseases (ICD-9 or ICD-10) diagnosis codes used by the CMS Chronic Conditions Warehouse project (14), in the preceding two years (15). Potential participants were excluded if they were non-English speaking, were mentally or physically incapable of completing the survey per caregiver report. All procedures performed in studies involving human participants were in accordance with the ethical standards of the Institutional Review Board and with the 1964 Helsinki declaration and its later amendments or comparable ethical standards. All study data were HIPAA-compliant and secured with additional password protection.

### Data Collection

Survey methods have been described previously (7). Briefly, using Alabama Medicaid enrollment and claims data files, the survey unit generated a list of potential participants who met the age, Medicaid enrollment and diabetes diagnosis inclusion criteria. We contacted potential participants by letter, which provided information about the study and an option to decline participation by contacting a toll-free number or by mail. Subsequently, study interviewers contacted potential participants who did not decline by phone to invite them to participate and schedule a time to complete the survey. Study interviewers called participants multiple times at different times and days, including evenings and weekends, with a maximum of 15 call attempts. For eligible participants who agreed to participate, informed consent was obtained by phone. Study interviewers used a computer-assisted telephone interview system to complete a 125-item survey which included measures detailed below. The study was reviewed and approved by the UAB Institutional Review Board.

### Measures

Measures (described below) included those that assessed self-reported health, depressive symptoms, perceived stress, diabetes management, and stress relating to diabetes with additional questions on socio-demographics. Participants during COVID completed additional questions relating to COVID experiences and access to care.

#### Self-Reported Health Survey

The self-reported health survey is a single-item ordinal measure with 5 levels varying from excellent, very good, good, fair, or poor. This survey is a widely used indicator of general health status in epidemiologic and population health research and is often categorized into excellent, very good, or good versus fair or poor (16).

#### Patient Health Questionnaire-8 (PHQ-8)

The PHQ-8 is an 8-item survey used for assessing depression that incorporates DSM-IV depression criteria with other leading major depressive symptoms into a numerical score graded as mild (5-9), moderate (10-14), moderately severe (15-19), and severe depression (>20) (17).

#### Perceived Stress Scale (PSS-4)

The PSS-4 is a 4-item survey used to measure perceived psychological stress relating to general stress in the previous month. Scores range from 0 to 16 with higher scores indicating greater perceived stress (18, 19).

#### Perceived Diabetes Self-Management Scale (PDSMS)

The PDSMS is an 8-item survey measuring diabetes self-efficacy, adapted from the Perceived Medical-Condition Self-Management Scale. Scores range from 8 to 40, with higher scores indicating more confidence in self-managing one’s diabetes. Higher scores are associated with higher reported self-care activities and better glycemic control (20).

#### Diabetes Distress Scale

The DDS is a 17-item scale that evaluates distress relating to the emotional burden, physician-related distress, regimen-related distress, and interpersonal distress of managing type 2 diabetes over the past month. The mean score is graded on a possible score range of 1–6 with a score of less than 2 indicating low diabetes distress, 2 to greater than 3 moderate diabetes distress and ≥3 severe diabetes distress (7).

#### Questions on the Impact of COVID

To assess the impact of COVID in the past 6-months, questions (n=20) were pulled from two sources and modified based on feedback from a community advisory board and/or to simplify administration over the phone (21, 22). Participants reported if they experienced any cancelled or rescheduled medical appointments, completed any telemedicine appointments, or faced any delay in diabetes medications or supplies. Participants were also asked if they experienced any income change *via* loss of employment or employment loss of spouse related to COVID.

### Analysis

**Figure 1:**
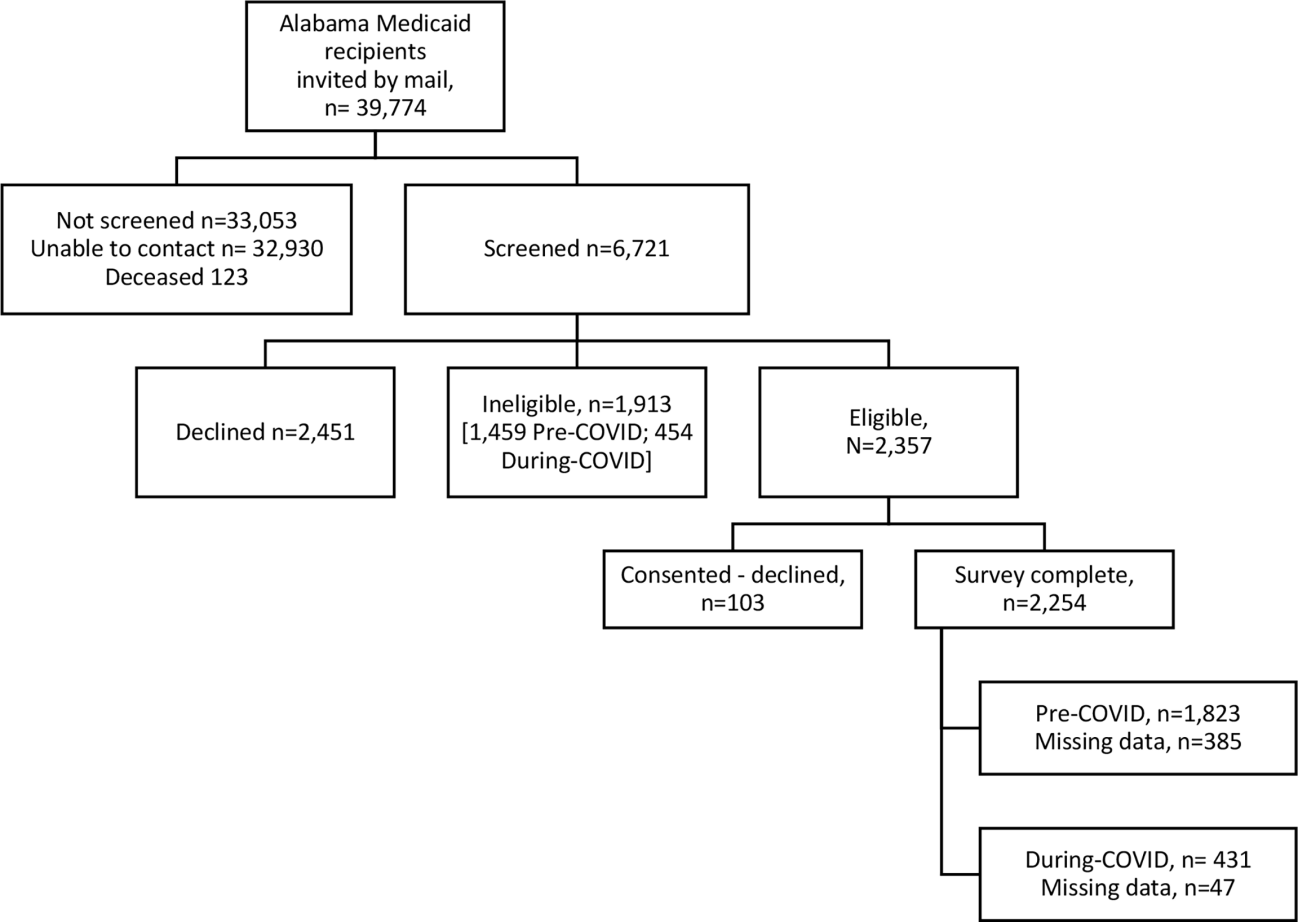
Survey population flow diagram of Alabama Medicaid-covered adults with type 2 diabetes mellitus (March 2017 to May 2021).

For analysis, participants were dichotomized into pre-COVID (March 2017 to October 2019) vs during-COVID (October 2020 to May 2021) groups (see **Figure 1**). These dates were defined based on availability of data. Survey implementation was paused after October of 2019 due to a Medicaid policy change whereby primary care providers were no longer designated as gatekeepers for care. Surveys were scheduled to resume in early Spring however this was delayed due to the impact of COVID on University operations; as such, survey implementation resumed in October 2020. All respondents were unique: no individuals were surveyed twice (pre and during COVID). First, we examined the distribution of continuous variables and the number of observations per cell. Descriptive statistics were used to characterize the study population overall and by COVID time period. Wilcoxon’s tests, t-tests, and chi-square tests were used to determine differences between demographics, depressive symptoms, perceived stress, diabetes distress and self-reported health between groups. Wilcoxon’s and t-test results were similar; results from Wilcoxon’s tests are presented here. Logistic regression was used to model the effect of COVID time period on self-rated health controlling for demographics, severity of diabetes, and diabetes distress. The self-reported health survey scores were dichotomized into to poor health (poor, fair) and better health (good, very good, excellent) for analysis. Covariates selected were either: 1. Specified *a priori* based on established or potential relationship of the covariate with our outcome variable; or 2. Found to differ between the pre- vs. during-COVID groups. Model 1 adjusted for demographics, model 2 added disease severity factors to model 1 and model 3 added diabetes distress to model 2. We present results for model 3. We then compared experiences of individuals reporting poor and better health during COVID.

## Results

### Population

In this sample of 1,822 individuals (**Table 1**), median age was 54, 74.5% were female and 59.4% were Black. Most participants had a high school level education and were unable to work. Median time with DM was 9 years and 44.2% were on insulin. Only 43.3% of participants reported having received diabetes education. Of the sample, 1,438 participants were dichotomized into pre-COVID (March 2017 to October 2019) group and 384 during-COVID (October 2020 to May 2021) group.

**Table 1:** Characteristics of survey participants with type 2 diabetes covered by Alabama Medicaid prior to the COVID-19 pandemic and during the COVID-19 pandemic.

Characteristic	ALL	Pre-COVID	During-COVID	P-value
	N = 1822	N = 1438	N = 384	
Age in years, median [IQR]	54 [45, 60]	55 [46, 60]	51 [42, 59]	<0.001
Sex, N (%)				<0.001
Male	465 (25.5%)	395 (27.5%)	70 (18.2%)	
Female	1357 (74.5%)	1043 (72.5%)	314 (81.8%)	
Race, N (%)				<0.01
White	703 (38.6%)	577 (40.1%)	126 (32.8%)	
Black	1083 (59.4%)	837 (58.2%)	246 (64.1%)	
Other	36 (2.0%)	24 (1.7%)	12 (3.1%)	
NonWhite, N (%)	1119 (61.4%)	861 (59.9%)	258 (67.2%)	<0.01
Hispanic, N (%)	54 (3.0%)	50 (3.5%)	4 (1.0%)	0.01
Education, N (%)				0.06
Less than HS	612 (33.6%)	499 (34.7%)	113 (29.4%)	
High School	735 (40.4%)	561 (39.1%)	174 (45.3%)	
More than HS	473 (26.0%)	376 (26.2%)	97 (25.3%)	
Income less than 10K, N (%)	1003 (67.4%)	783 (67.6%)	220 (66.9%)	0.81
Employment, N (%)				<0.001
Working/Studying	107 (5.9%)	73 (5.1%)	34 (8.9%)	
Not working	184 (10.1%)	111 (7.8%)	73 (19.1%)	
Cannot work	1524 (84.0%)	1248 (87.2%)	276 (72.1%)	
Marital Status, N (%)				0.38
Single	1448 (79.5%)	1149 (79.9%)	299 (77.9%)	
Married	374 (20.5%)	289 (20.1%)	85 (22.1%)	
Rural, N (%)	664 (36.4%)	552 (38.4%)	112 (29.2%)	<0.001
Diabetes Duration in years, median [IQR]	9[3,18]	9[4,18]	7[2,16]	<0.001
Insulin Use, N (%)	806 (44.2%)	643 (44.7%)	163 (42.4%)	0.43
Diabetes Education, N (%)	788 (43.4%)	612 (42.7%)	176 (46.0%)	0.25
***Psychosocial Measures***				
Self-Reported Health				0.12
Better	919 (50.4%)	739 (51.4%)	180 (46.9%)	
Poor	903 (49.6%)	699 (48.6%)	204 (53.1%)	
PHQ-8 Scale, median [IQR]^a^	6 [3, 10]	6 [3, 11]	5 [2, 10]	0.14
PDSMS Scale, median [IQR]^b^	28 [25, 32]	28 [25, 31]	30 [26, 32]	<0.001
PSS4, median [IQR]^c^	5 [2, 8]	6 [2, 8]	5 [2, 7]	<0.01
DDS, median [IQR]	1.3 [1.1, 1.8]	1.4 [1.1, 1.9]	1.2 [1.1, 1.6]	<0.01

Patient Health Questionnaire-8 (PHQ-8), Perceived Diabetes Self-Management Scale (PDSMS), Perceived Stress Scale (PSS4), Diabetes Distress Scale (DDS).

^a^ N=14 were missing from the Pre-COVID group, ^b^ N=16 were missing (12 from Pre-COVID and 4 from During-COVID); ^c^ N=62 were missing (61 from Pre-COVID and 1 from During-COVID).

### Bivariate Associations by COVID Time Group

Participants during COVID were younger with higher proportion of Black individuals (64.1% VS 58.2%, p=0.01) and more females (81.8% VS 72.5%, p<0.001). The during COVID group had fewer individuals reporting inability to work (72.1% VS 87.2%, p<0.001); fewer individuals from rural areas (29.2% VS 38.4%, p<0.001); and a shorter diabetes duration (10.6 years VS 12.5 years, p<0.001). The during COVID group reported slightly higher perceived self-management (PDSMS Score 30 VS 28, p<0.001); moderately better stress ratings (PSS-4 Score 5 VS 6, p<0.01); and modestly lower levels of diabetes distress (1.4 VS 1.2, p<0.01) when compared to the pre-COVID group.

### Multivariable Analysis

After adjusting for demographic differences, diabetes severity, and diabetes distress, participants responding during COVID had increased odds of reporting poor or fair health (Odds ratio [OR] 1.41, 95% Confidence Interval [CI] 1.11-1.80) as seen in **Table 2**.

**Table 2:** Association of COVID time period on self-reported poor health among survey participants with type 2 diabetes covered by Alabama Medicaid (N = 1,822).

Characteristic	Comparison	Odd Ratio (95% Confidence Limits)	P-value
COVID time period	Pre-COVID vs During-COVID	1.41 (1.11-1.8)	<0.01
Age (years)	Per one-year increase	1.00 (0.99-1.01)	0.87
Gender	Male vs Female	0.78 (0.62-0.98)	0.03
Race	Nonwhite vs White	0.66 (0.53-0.81)	<.0001
Education	Less than High School vs More than High School	1.23 (1-1.52)	0.05
Marital Status	Married vs Not Married	0.85 (0.67-1.09)	0.21
Rurality	Rural vs Non-rural	1.04 (0.84-1.27)	0.74
Diabetes Duration (years)	Per one-year increase	1.00 (0.99-1.01)	0.67
Insulin use	Yes vs No	1.17 (0.95-1.43)	0.15
Diabetes Distress Scale (score)	Per one-point increase	2.71 (2.29-3.22)	<.0001

### Bivariate Differences Between Poor Health vs Better Health During COVID

Participants during COVID who reported poor health were less likely to be working (5.9% VS 12.3%, p=0.0352) compared to those with better health. This group was also less likely to be married (81.9% VS 73.3%, p=0.0445). This group had higher PHQ-8 scores (8 VS 3, p<0.001), lower PDSMS scores (28 VS 30, p<0.001), higher PSS-4 scores (5 VS 4, p<0.001), and higher DSS scores (1.2 VS 1.4, p<0.001) as seen in **Table 3**.

**Table 3:** Comparison of during-COVID survey participants with type 2 diabetes mellitus by self-reported health status.

Characteristic	Total	Better health	Poor Health	P-value
	N = 384	N = 180	N = 204	
Age in years, median [IQR]	51 [42, 59]	52 [42, 59]	51 [41, 58]	0.28
Sex, N (%)				0.40
Male	70 (18.2%)	36 (20.0%)	34 (16.7%)	
Female	314 (81.8%)	144 (80.0%)	170 (83.3%)	
Race, N (%)				0.67
White	126 (32.8%)	55 (30.6%)	71 (34.8%)	
Black	246 (64.1%)	119 (66.1%)	127 (62.3%)	
Other	12 (3.1%)	6 (3.3%)	6 (2.9%)	
Nonwhite, N (%)	258 (67.2%)	125 (69.4%)	133 (65.2%)	0.38
Hispanic, N (%)	4 (1.0%)	2 (1.1%)	2 (1.0%)	0.90
Education, N (%)				0.19
Less than HS	113 (29.4%)	45 (25.0%)	68 (33.3%)	
High School	174 (45.3%)	88 (48.9%)	86 (42.2%)	
More than HS	97 (25.3%)	47 (26.1%)	50 (24.5%)	
Income less than 10K, N (%)	220 (66.9%)	95 (63.8%)	125 (69.4%)	0.28
Employment, N (%)				0.04
Working/Studying	34 (8.9%)	22 (12.3%)	12 (5.9%)	
Not working	73 (19.1%)	38 (21.2%)	35 (17.2%)	
Cannot work	276 (72.1%)	119 (66.5%)	157 (77.0%)	
Married, N (%)	85 (22.1%)	48 (26.7%)	37 (18.1%)	0.05
Rural, N (%)	112 (29.2%)	53 (29.4%)	59 (28.9%)	0.91
Diabetes Duration, median [IQR]	7[2,16]	9[2,17]	7[2,16]	0.26
Insulin Use, N (%)	74 (41.1%)	89 (43.6%)	163 (42.4%)	0.62
Diabetes Education, N (%)	80 (44.7%)	96 (47.1%)	176 (46.0%)	0.64
***Psychosocial Measures***				
PHQ-8 Scale, median [IQR]^a^	5 [2, 10]	3 [1, 7]	8 [4, 13]	<0.001
PDSMS Scale, median [IQR]^b^	30 [26, 32]	30 [27, 32]	28 [24, 32]	<0.01
PSS4, median [IQR]	5 [2, 7]	4 [1, 6]	5 [3, 8]	<0.001
DDS, median [IQR]^a^	1.2 [1.1, 1.6]	1.2 [1.0, 1.4]	1.4 [1.1, 1.9]	<0.001
				

Patient Health Questionnaire-8 (PHQ-8), Perceived Diabetes Self-Management Scale (PDSMS), Perceived Stress Scale (PSS4), Diabetes Distress Scale (DDS).

^a^ n=4 missing (1 from Better Health and 3 from Poor Health); ^b^ n=1 missing (1 from Poor Health).

### Impact of COVID Between Poor Health vs Better Health During COVID

Among participants surveyed during COVID, 34.8% of participants experienced an appointment cancellation or rescheduled appointment with majority due to doctor’s office canceling or moving appointment. Over half of participants were offered a virtual visit *via* telehealth or phone visit with majority of this group completing a telephone visit. Within this group, 19.8% of participants reported a video visit. Participants who reported poor health were significantly more likely to believe they have had COVID-19 regardless of testing.

## Discussion

We found significant differences between the pre-COVID and during-COVID groups participants (**Table 1**). Notably, the during-COVID group is a younger and more urban group with less diabetes severity. This likely contributes to the higher levels of diabetes self-management (PMDS), reduced perceived stress (PSS-4), and lower levels of diabetes distress (DDS) observed in this analysis. However, there was a modest decrease in self-reported health in the during-COVID group. Multivariate analysis allowed a deeper look into the association of the COVID time period and poor self-reported health (**Table 2**). After controlling for demographics, severity of diabetes, and diabetes distress, the participants surveyed during COVID were significantly more likely to report poor self-reported health compared to those pre-COVID. Within the during-COVID group, individuals reporting poor health were more likely to report worse depressive symptoms, worse perceived stress, less competency in managing their diabetes, and higher levels of diabetes distress compared to those with better health as seen in **Table 3**. These factors likely contribute to an overall decreased sense of health and could contribute to worse health outcomes in this group.

Our survey questions related to healthcare experiences during COVID (**Table 4**) shed additional light on possible barriers experienced by participants. Over a third of respondents experienced cancellation or rescheduled appointment and more than half experienced a virtual health appointment. However, few participants reported delays of care or experienced changes to household income during COVID. Moreover, there was no significant difference in experience between individuals reporting better health or poor health. We predicted that this transition may have been challenging to populations with significant financial barriers and limited access to or unfamiliarity with technology (10, 11, 23). However, this population tolerated this transition well and seems to have minimal changes to their care and household income. This could be related to a limited baseline utilization of healthcare resources within this population prior to COVID and the increased use of virtual medicine during COVID. Recent studies describing the many benefits of virtual care suggest that virtual care is effective in managing patients with diabetes, especially in low socioeconomic groups. With proper access, virtual care can motivate patients, facilitate better medical competency, and provide better decision support for patients. These studies also support the effectiveness of virtual care shown by A1C management similar to or superior to usual care (24).

**Table 4:** Responses to COVID specific questions by self-reported health status among survey participants who participated during COVID (October 2020 to May 2021).

Survey item	Total (N=384)	Better health (n=180)	Poor Health (n=204)	P-value
Cancelled health-related appointments or treatments	133 (34.8%)	56 (31.3%)	77 (37.9%)	0.17
Rescheduled or postponed any health-related appointments or treatments	146 (38.2%)	67 (37.2%)	79 (39.1%)	0.71
Provider offered a video visit or phone visit	207 (53.9%)	96 (53.3%)	111 (54.4%)	0.83
Completed a telephone visit	195 (50.8%)	89 (49.4%)	106 (52.0%)	0.62
Completed a video visit	76 (19.8%)	33 (18.3%)	43 (21.1%)	0.50
Delayed in needed diabetes medicines	39 (11.5%)	15 (9.6%)	24 (13.1%)	0.30
Delayed in needed diabetes testing supplies	45 (11.8%)	18 (10.1%)	27 (13.3%)	0.34
Due to the coronavirus pandemic, my household income has:				0.75
Decreased	71 (18.6%)	36 (20.2%)	35 (17.2%)	
Increased	30 (7.9%)	14 (7.9%)	16 (7.9%)	
Not changed	280 (73.5%)	128 (71.9%)	152 (74.9%)	
Lost job or primary source of income due to COVID	19 (5.0%)	12 (6.7%)	7 (3.4%)	0.14
Spouse or partner lost job or primary source of income due to COVID	14 (3.7%)	5 (2.8%)	9 (4.5%)	0.40
Obtained COVID19 testing	227 (59.1%)	106 (58.9%)	121 (59.3%)	0.93
Received Positive COVID test	43 (19.0%)	22 (21.0%)	21 (17.4%)	0.49
Believe you have had COVID-19	92 (24.3%)	35 (19.8%)	57 (28.4%)	0.05

Participants were asked if they experienced the above over the last 6 months.

Interestingly, participants who reported poor health were significantly more likely to believe they have had COVID regardless of testing. Additional studies may be needed to identify other contributors to poor self-reported health during COVID within this Alabama Medicaid population.

### Limitations

This is a cross-sectional study; thus, we are unable to infer causation between COVID and poor self-reported health. A longitudinal survey with the same respondents at both time periods would help mitigate this limitation. Further, we cannot be sure that differences were not due to policy changes occurring within Medicaid itself or other seasonal changes that may have co-occurred temporally. Our study sample includes adults with type 2 diabetes covered by Alabama Medicaid, which limits the generalizability of our findings to other populations. We used single item measures to review self-reported health which does not fully reflect the multidimensionality or complexity of a participant’s health. Assessments of health were based on self-report, we were unable to include objective measures of diabetes control such as HbA1c. Additional limitations include the limited number of respondents in the during-COVID group and significant demographic differences between respondent groups.

### Conclusions

In this sample of Medicaid-covered adults with type 2 DM living in Alabama, individuals surveyed during-COVID reported poorer health compared to those surveyed pre-COVID. Within the during-COVID group, those reporting poor health were more likely to report worse psychosocial symptoms than those who reported better health. Further findings also suggest that while individuals reporting poor health and better health faced similar delays in care, those reporting poor health during-COVID were more likely to believe they have had COVID regardless of testing. Taken together, these findings suggest that physical and mental health may have worsened among some low-income individuals with type 2 DM. Outreach efforts should include specific screening for stress and depression in order to identify and support those in need.

## Data Availability Statement

The datasets presented in this article are not readily available because of institutional restriction, the generated dataset is not publicly available. Requests to access the datasets should be directed to Andrea Cherrington, MD, MPH, acherrington@uabmc.edu.

## Ethics Statement

The studies involving human participants were reviewed and approved by University of Alabama at Birmingham, Office of the Institutional Review Board. Written informed consent for participation was not required for this study in accordance with the national legislation and the institutional requirements.

## Author Contributions

ACA contributed to the design of the study, analyzed the data, wrote the manuscript, and revised prior drafts of the manuscript for intellectually important content. CH contributed to the design of the study, wrote the manuscript, and revised prior drafts of the manuscript for intellectually important content. ACA, CH, and CP assisted LJ with the analysis and revised the manuscript for intellectually important content. EL contributed to the conception and design of the study, provided expertise on the analysis plan, and revised the manuscript for intellectually important content. AAA contributed to the design of the study and provided expertise on survey implementation. LJ takes responsibility for the integrity of the data and the accuracy of the data analysis. CP provided input on the analysis plan and revised prior drafts of the manuscript for intellectually important content. ACA and AC conceived and designed the study, assisted in data analysis and interpretation, wrote parts of the introduction and discussion section, and revised prior drafts of the manuscript for intellectually important content. The survey data from reported here were collected from Alabama Medicaid Agency recipients with diabetes mellitus. All authors contributed to the article and approved the submitted version.

## Funding

The project described was supported by the National Institute of Diabetes and Digestive and Kidney Diseases (R18DK109501, Cherrington). Support was also provided by UAB Diabetes Research Center (P30 DK079626, Cherrington).

## Author Disclaimer

The content is solely the responsibility of the authors and does not necessarily represent the official views of the National Institute of Diabetes and Digestive and Kidney Diseases, the National Institutes of Health, or the Alabama Medicaid Agency. Representatives for the Alabama Medicaid Agency were provided an opportunity to review and provide comments on the manuscript prior to submission. This manuscript was not prepared in collaboration with the Alabama Medicaid Agency and does not necessarily reflect the opinions or represent the official views of the Alabama Medicaid Agency.

## Conflict of Interest

The authors declare that the research was conducted in the absence of any commercial or financial relationships that could be construed as a potential conflict of interest.

## Publisher’s Note

All claims expressed in this article are solely those of the authors and do not necessarily represent those of their affiliated organizations, or those of the publisher, the editors and the reviewers. Any product that may be evaluated in this article, or claim that may be made by its manufacturer, is not guaranteed or endorsed by the publisher.
